# Infection-Induced Vulnerability of Perinatal Brain Injury

**DOI:** 10.1155/2012/102153

**Published:** 2011-11-02

**Authors:** Carina Mallard, Xiaoyang Wang

**Affiliations:** ^1^Department of Neuroscience and Physiology, Sahlgrenska Academy, University of Gothenburg, P.O. Box 432, 40530 Göteborg, Sweden; ^2^Department of Pediatrics, The Third Affiliated Hospital of Zhengzhou University, Zhengzhou 450052, China

## Abstract

A growing body of evidence demonstrates that susceptibility and progression of both acute and chronic central nervous system disease in the newborn is closely associated with an innate immune response that can manifest from either direct infection and/or infection-triggered damage. A common feature of many of these diseases is the systemic exposure of the neonate to bacterial infections that elicit brain inflammation. In recent years, the importance of innate immune receptors in newborn brain injury, the so-called Toll-like receptors, has been demonstrated. In this paper we will discuss how neonatal sepsis, with particular emphasis on *Escherichia coli*, coagulase-negative staphylococci, and group B streptococcal infections in preterm infants, and Toll-like receptor-mediated inflammation can increase the vulnerability of the newborn brain to injury.

## 1. Introduction

Perinatal brain injury represents a significant clinical problem [1]. A growing body of evidence demonstrates that susceptibility and progression of both acute and chronic central nervous system (CNS) disease is closely associated with an innate immune response that can manifest from either direct infection and/or infection-triggered damage [2]. A common feature of these diseases is the systemic activation of inflammatory mediators, which via the blood can disrupt the blood-brain barrier, affect the circumventricular organs in the brain (which lack a blood-brain barrier), or interact with the brain endothelium, thereby eliciting brain inflammation [3]. Furthermore, the presence of activated inflammatory cells derived from systemic circulation or from dormant brain resident populations is a key feature of many CNS diseases. More recently, the importance of innate immune receptors in CNS injury, the so-called Toll-like receptors (TLRs), has also been emphasized. In this paper we will focus on how neonatal sepsis and TLR-mediated inflammation increase the vulnerability of the newborn brain. 

## 2. Neonatal Sepsis and Brain Injury

Infants with sepsis have an increased incidence of cerebral palsy [4] and white matter abnormalities [5–11]. In a large study of 6093 extremely low birth weight (<1000 g) infants, those who were infected (including early-onset sepsis, suspected sepsis (culture negative), and had necrotizing enterocolitis (NEC)) were more likely to have cerebral palsy than children who did not have a neonatal infection [12]. In another recent large sample-size study involving 1155 infants born at 23 to 27 weeks gestation, it was found that children who had both late bacteremia (positive blood culture result after the first postnatal week) and surgical NEC were at increased risk of diparetic cerebral palsy compared with children who had neither [13]. Moreover, by comparing outcomes of 150 infants with periventricular leukomalacia (PVL) with controls matched for gestational age, it was found that infants with bacterial sepsis were twice as likely to develop PVL, and those with meningitis were almost four times as likely to develop white matter disease [14]. Similar findings were noted in a smaller case-control study, where associations between cerebral palsy, clinical chorioamnionitis and sepsis were demonstrated [15]. Moreover, there was an increased incidence of Gram-negative bacterial and fungal infections in a very low birth weight population, and these infants were at significantly increased risk for moderate to severe cerebral palsy and neurodevelopmental impairment at 18 months of age [16]. 

### 2.1. Bacterial Pathogens in Neonatal Sepsis

*Escherichia coli* is one of the main pathogens causing early-onset infections in preterm neonates, accounting for up to 40% of the cases of bacteremia among very low birth weight preterm infants (<1,500 g) [17]. Cerebral white matter injury has been found by MRI following *Escherichia coli* meningitis in human newborn infants [18]. Furthermore, *Escherichia coli* induce brain damage in a number of antenatal rabbit and rodent models [19–26]. Also, in a recent study, white matter injury was demonstrated in an animal model of neonatal *Escherichia coli* sepsis in 5-day-old rat pups [27]. Experimental studies show that early-life *Escherichia coli* exposure can also have long-term effects, influencing the vulnerability to other factors in adulthood, for example, age-related cognitive decline [28] as well as attenuated glial and cytokine responses to amphetamine challenge [29]. 

In recent years, coagulase-negative staphylococci (CONS) have emerged as the most prevalent and important neonatal pathogens, responsible for approximately 50% of all episodes of late-onset neonatal sepsis in neonatal intensive care units around the world [30–33]. CONS cause significant morbidity, mortality, and healthcare costs worldwide in preterm newborns, especially in very low birth weight infants [34–38]. The vulnerability of preterm infants to CONS infection has been suggested to be due to the special characteristics of the premature infant's innate immunity [39]. Although there is no direct evidence of CONS causing perinatal brain injury, the presence of CONS in the chorioamnion space at delivery is associated with increased risk for the development of cerebral palsy in preterm infants [40, 41]. Further, in children with an established diagnosis of cerebral palsy, who are admitted to pediatric intensive care, there is a high rate of carriage of abnormal bacteria, including CONS [42]. 

In very low birth weight preterm infants with early onset neonatal sepsis, the rate of group B streptococcal (GBS) infections is relatively low in comparison with *E. coli* infections [17]. There is no direct evidence of GBS sepsis playing a role in cerebral palsy; however, nearly half of all infants who survive an episode of GBS meningitis suffer from long-term neurodevelopmental sequelae [43]. Further, extensive cortical neuronal injury was found in GBS-infected neonatal rats, which was mediated through reactive oxygen intermediates [44, 45]. 

## 3. Toll-Like Receptor-Mediated Vulnerability of the Immature Brain

### 3.1. Toll-Like Receptors

**Figure 1:**
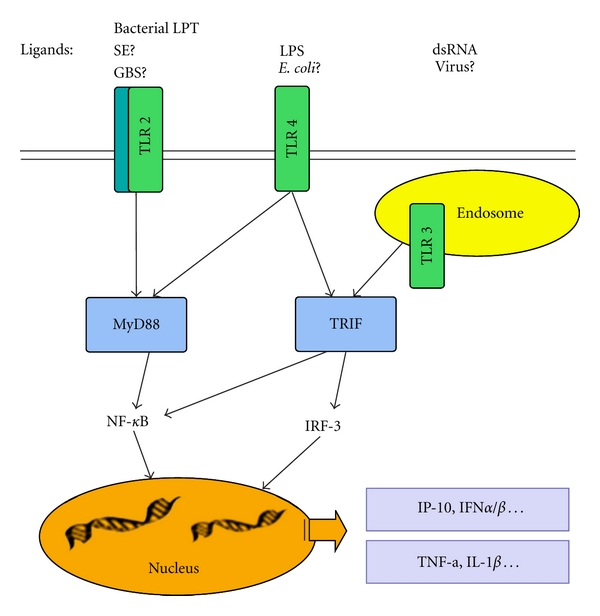
Diagram outlining infectious agents, TLRs, and major signaling pathways. Abbreviations: SE: S. epidermidis; GBS: group B streptococcus; LPT: lipopeptides. LPS: lipopolysaccharide; MyD88: myeloid differentiation primary response gene (88); TRIF: TIR domain-containing adaptor inducing interferon-*β*-mediated transcription factor; NF-*κ*B: nuclear factor-KappaB; IRF: interferon regulatory factor; IP-10: interferon gamma-induced protein 10; IFN: interferon; TNF: tumor necrosis factor; IL-1: Interleukin -1.

Toll-like receptors (TLRs) play a central role in primary recognition of infectious and viral pathogens. The presence of all 13 known TLRs has been demonstrated in the brain [46–48]. TLR4 mediates cellular activation in response to LPS derived from *Escherichia coli* [49], while CONS [39] and GBS infections [50] are, at least partly, believed to be mediated by TLR2. Interestingly, the role of TLRs in nonbacterial-induced brain injury has also recently been highlighted [51]. TLRs signal through the recruitment of intracellular adaptor proteins, followed by activation of protein kinases and transcription factors that induce the production of inflammatory mediators (Figure 1). The adaptor protein MyD88 is used by most TLRs, except TLR3, while the TRIF adaptor protein is used only by TLR3 and TLR4. LPS-induced activation of TLR4 elicits, via both MyD88 and TRIF, a broad inflammatory response in tissues, including the immature brain [52]. 

### 3.2. TLR Expression during Brain Development

There is relatively little information regarding the expression of TLRs in the developing brain. During embryonic life, protein expression of both TLR-3 and -8 has been identified [53, 54], while TLR-2 expression is relatively low before birth and increases during the first two weeks of life [55]. We have shown that mRNA for TLR1-9 is expressed in the neonatal mouse brain [56]. It appears that some of the TLRs may play important roles during normal brain development, as TLR2 inhibits neural progenitor cell proliferation during the embryonic period, and TLR3 deficiency increases proliferation of neural progenitor cells, while TLR8 stimulation inhibits neurite outgrowth [53–55]. In support, TLR2 and TLR4 have been shown to regulate hippocampal neurogenesis in the adult brain [57].

### 3.3. LPS-Induced Brain Injury

We, and others, have shown that systemic administration of LPS results in brain injury in both fetal and newborn animals [58–60]. These injuries appear, both histologically and by MRI analysis, to be very similar to those found in preterm infants [61]. Furthermore, it is now well established that pre-exposure to LPS can increase the vulnerability of the immature brain to hypoxia-ischemia (HI), in both rats [62, 63] and mice [64]. These effects are TLR4 [65] and MyD88 dependent [64, 66]. In a recent study, it was also shown that a very low dose of LPS, specifically increased the vulnerability of the immature white matter [67]. Low-dose LPS (0.05 mg/kg) sensitized HI injury in P2 rat pups by selectively reducing myelin basic protein expression and the number of oligodendrocytes while increasing neuroinflammation and blood-brain barrier damage in the white matter. The neuroinflammatory responses to LPS/HI appears to be age dependent [68]. Rat pups subjected to LPS/HI at P1 responded with weak cytokine response, while there was a prominent upregulation of cytokines in P12 pups subjected to the same insult. Interestingly, IL-1*β* was upregulated at both ages; IL-1*β* injections sensitize the newborn brain to excitotoxicity [69] and repeated IL-1*β* exposure during the neonatal period induces preterm like brain injury in mice [70].

Although it has clearly been demonstrated that LPS can increase the vulnerability to HI, under certain circumstances LPS can also induce tolerance to brain injury. We have shown that the time interval between LPS exposure and the subsequent HI is imperative to the outcome [71, 72], where a 24 h interval seems to induce a tolerant state that makes the brain less vulnerable. This has been confirmed by others who have implicated several possible mechanisms, including upregulation of corticosterone [73], which is further supported by the fact that administration of dexamethasone prevents learning impairment following LPS/HI in neonatal rats [74]. Furthermore, Akt-mediated eNOS upregulation in neurons and vascular endothelial cells have been implicated in LPS-induced preconditioning [75]. 

The importance of the time interval between LPS and other insults seems to be a generalized phenomenon. We have recently demonstrated in an in vitro model that conditioned medium from LPS-activated microglia affects the antioxidant Nrf2 system and cell survival in astrocytes in a time-dependent manner. LPS-induced inflammation had dual, time-dependent, effects on the Nrf2 system in that sustained activation (72 h) of GSK3beta and p38 downregulated the Nrf2 system, possibly via the activation of histone deacetylases, changes that were not observed with a 24 h (tolerance) interval [76, 77]. These studies support our previous report demonstrating that reductions in antioxidants were more pronounced when HI was preceded by LPS injection in 8-day rats 3 days prior to the HI insult [78].

### 3.4. Other TLRs in Perinatal Brain Injury

Compared to TLR4, much less is known about other TLRs in perinatal brain injury. As mentioned above, TLR2, TLR3, and TLR8 can affect normal brain development [53–55]. Activation of TLR2 in neonatal mice decreases volume of cerebral gray matter, white matter in the forebrain, and cerebellar molecular layer [79]. Further, we have recently demonstrated the expression of both TLR1 and TLR2 in the neonatal mouse brain following HI. In these studies, TLR2 deficiency resulted in reduced infarct volume after HI, while TLR-1-deficient mice were not protected [56]. 

Maternal viral immune activation is believed to increase the risk of psychiatric disorders such as schizophrenia in offspring, and in order to examine this relationship, several authors have investigated the vulnerability of the fetal brain to synthetic double-stranded RNA, polyriboinosinic-polyribocytidilic acid (poly I:C), a TLR3 agonist. Maternal injection with poly I:C towards the end of gestation (≥G15) causes sensorimotor gating deficits in the adult offspring in mice [80] and increased sensitivity to the locomotor-stimulating effects of MK-801 [81]. The effects of Poly I:C appear to be gestational age dependent [82]. Maternal Poly I:C injection on GD9, but not GD17, significantly impaired sensorimotor gating and reduced prefrontal dopamine D1 receptors in adulthood, whereas prenatal immune activation in late gestation impaired working memory, potentiated the locomotor reaction to a NMDA-receptor antagonist, and reduced hippocampal NMDA-receptor subunit 1 expression. In particular, Poly I:C injections early during rodent pregnancy affect structural brain development, such as a transient decrease of myelin basic protein in the neonatal offspring [83] and cerebellar pathology [84].

## 4. Conclusion

*E. coli* infections are common in preterm neonates, and considerable evidence suggests that *E. coli*-induced inflammation play a role in the development of white matter damage in preterm infants. There is much less data available concerning the importance of two other common neonatal pathogens, CONS and GBS, in perinatal brain injury. Furthermore, it is becoming clear that TLRs have important roles during development and may be involved in both pathogen-induced damage as well as so called “sterile” HI-induced inflammation. In order to better understand the underlying causes of perinatal brain injury, the interaction between common neonatal pathogens and TLRs in the newborn brain deserves further investigation.
